# Solid-State Precipitation of Silver Nanoparticles Nucleated during Al Anodizing: Mechanism and Antibacterial Properties

**DOI:** 10.1021/acsabm.4c01694

**Published:** 2025-01-28

**Authors:** Teo Atz-Dick, Renato de Castro Valente, Thiago Vignoli Machado, Fabiana Horn, Luís F. P. Dick

**Affiliations:** † Laboratório de Processos Eletroquímicos e Corrosão-ELETROCORR, Departamento de Metalurgia, Universidade Federal do Rio Grande do Sul, Avenida Bento Gonçalves 9500, 91501-970 Porto Alegre, Brazil; ‡ Departamento de Biofísica, Universidade Federal do Rio Grande do Sul, Avenida Bento Gonçalves 9500, 91501-970 Porto Alegre, Brazil

**Keywords:** antibacterial surface, Ag–Al, anodizing, Ag nanoparticles, Escherichia coli

## Abstract

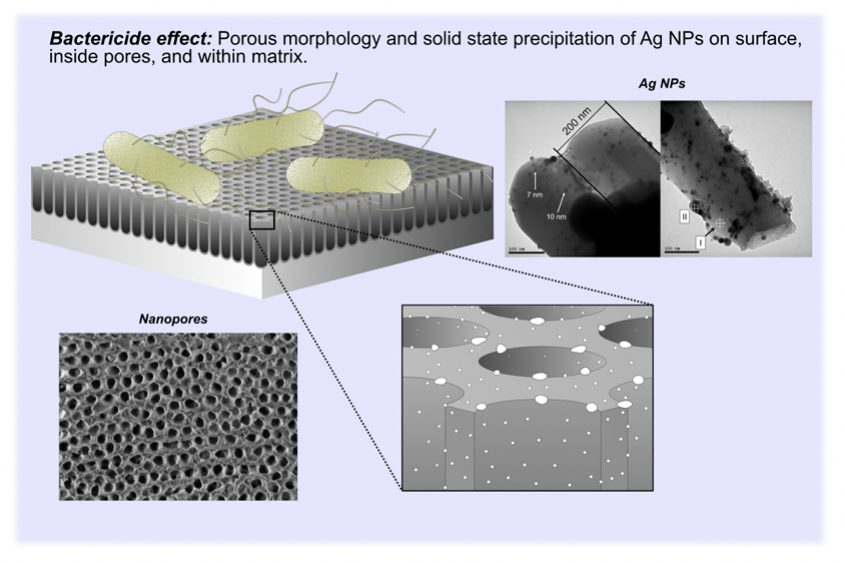

This study presents an innovative approach to creating antibacterial aluminum surfaces by combining the antibacterial properties of silver nanoparticles (Ag NPs) with the nanoarchitecture of anodized aluminum oxide in one step. An Al–Ag alloy containing 10 wt % Ag was synthesized and anodized in 0.3 M oxalic acid. Ag NPs precipitated in the solid state during anodization, resulting in a porous nanocomposite structure. Comprehensive characterization using SEM, TEM, and EDS revealed a 43 μm thick oxide layer with uniformly distributed nanopores of approximately 100 nm in diameter. Ag NPs with diameters ranging from 2 to 14 nm precipitated dispersed on the surface, inside pores, and within the Al_2_O_3_ matrix. Antibacterial properties were evaluated against *Escherichia coli*. The anodized Al–Ag surface demonstrated robust antibacterial activity after short incubation times (up to 1 × 10^8^ CFU/ml after 3 h). The enhanced antibacterial properties are attributed to the optimal size and distribution of Ag NPs and the potential physical bactericidal effect of the nanoporous structure. This strategy for the precipitation of Ag NPs in the solid state could be used to fabricate high-touch surfaces in hospitals.

## 1 Introduction

Aluminum (Al) alloys are essential for the fabrication of a wide range of nonimplantable medical devices and surfaces in the hospital setting due to their corrosion resistance and strength-to-weight ratio. However, when frequently touched by healthcare providers and patients, Al surfaces can develop long-lasting contamination by bacteria (high-touch surfaces) and become significant sources of hospital-acquired infections.^1−3^ The initial attachment of bacteria to these surfaces is influenced by several surface properties, such as charge, composition, stiffness, roughness, chemical composition, and hydrophobicity/hydrophilicity, which are essential to the initial bacterial attachment.^4^ Therefore, optimizing the surface properties of Al to avoid initial bacterial attachment and proliferation is crucial for reducing morbidity related to hospital-acquired infections.

Deposition of silver nanoparticles (Ag NPs) on a surface is a well-established strategy for improving their antibacterial properties. The bactericidal mechanism of Ag NPs involves the release of Ag^+^ ions, which generate reactive oxygen species, interacting with bacterial membranes and proteins and disrupting cellular functions.^5^ Ag NPs exhibit enhanced antibacterial efficacy compared to bulk silver due to their increased surface area-to-volume ratio, higher solubility, and potential for cellular internalization.^5^ Several strategies to incorporate Ag NPs on surfaces have been developed. Some authors added Ag NPs to polymeric films that could be applied over surfaces,^6,7^ while others performed chemical strategies to deposit Ag NPs on top of surfaces.^8,9^ While both approaches resulted in successful bactericidal effects, they might not be appropriate for high-touch hospital surfaces due to the constant cleaning with aggressive solutions and wear by long-term use.^3,10^ Accordingly, there could be benefits for antibacterial properties in developing processes capable of incorporating Ag NPs into metal matrices, compared to the deposition of nanoparticles on the material surface.

More recently, studying antibacterial properties through nanoarchitecture topographies has received increased attention. Some refer to this approach as bioinspired because many naturally occurring surfaces with high aspect-ratio topographies have been discovered to exhibit antibacterial properties. Examples are insects’ wings from cicadas and dragonflies, which contain nanopillars on their surfaces.^11,12^ The biocide mechanism involved is solely physical, by which surface tension and topography contribute to the mechanical disruption of the cell membrane.^12−15^ Employing this physical strategy on material surfaces can result in significant advantages over traditional methods using antibacterial agents or other chemical processes, as antibacterial resistance and the introduction of toxic substances into the body are avoided. On Al, nanotopographies capable of the same physical bactericide mechanism can be achieved through anodizing.^16,17^ This electrochemical technique can be used to fabricate nanostructured surfaces with precise control over morphology by manipulating various process parameters, including current density, electrolyte concentration and composition, and treatment duration.^18^

For applying Al in high-touch surfaces, a combination of the two approaches mentioned above, generating nanopores through anodizing and using Ag NPs, could be highly promising. However, the available strategies in the literature for incorporating Ag NPs rely on depositing Ag on top of nanopores, which demands extra steps after anodization and generates a surface that could be vulnerable to the removal of Ag NPs by mechanical or chemical processes. Ideally, Ag NPs should be contained on top of the anodic porous layer, inside pores, and within the anodic layer as a composite for a long-lasting antibacterial layer. In this work, this was achieved in one step by an innovative Ag NP synthesis strategy in the solid-state during the anodization of Al. First, Ag was added to Al to form a metastable solid solution. Then, through anodization, the conditions for Ag NPs to precipitate in the solid state were given. The result is a nanoporous Al_2_O_3_ layer with embedded Ag NPs. A comprehensive material characterization study was performed, proposing a mechanism for the precipitation of Ag NPs. Additionally, preliminary biological tests using *Escherichia coli* revealed a robust antibacterial effect. The proposed solid-state Ag NP precipitation reaction could be a promising strategy for fabricating materials for high-touch surfaces in healthcare settings.

## 2 Methods

### 2.1 Alloy Synthesis

The cast Al–Ag alloy with 10 wt % (3 at. %) was prepared with Al rods (Sigma-Aldrich 99.999% Al) and Ag pellets (Sigma-Aldrich 99.9% Ag) by fusion in a quartz alumina crucible under 10^–3^ mbar vacuum. The cast alloy was rolled in a hand mill to obtain a thickness of 0.5 mm. Then, the cast samples were solubilized to dissolve Ag crystals, and Ag_2_Al precipitates at 560 °C for 6 h in a 10^–3^ mbar vacuum and rapidly quenched in water to avoid the precipitation of the δ phase (Ag_2_Al). The precipitation of Guinier–Preston zones and coherent δ-Ag_2_Al depends on the slow solid-state diffusion of Ag and is thus also avoided. After solubilization, the samples were cut to size (1 cm^2^), sanded, and polished to 1 μm with diamond paste. Finally, the samples were degreased by sonicating in isopropanol, rinsing in deionized water, and drying in a hot air flux.

### 2.2 Anodizing

The samples were anodized in galvanostatic mode with a current density of 50 mA/cm^2^ in 0.3 M oxalic acid for 15 min at room temperature. The anodizing transients were plotted as a function of the potential applied for constant current maintenance. Immediately after anodization, the samples were rinsed several times in distilled water to remove oxalic acid in the pores, followed by isopropanol, and dried.

### 2.3 Scanning Electron Microscopy

A field emission gun scanning electron microscope (FEG SEM Zeiss Auriga) with an integrated electron dispersion spectrometer was used to study the morphology of the anodic oxide, with secondary and backscattered electron imaging. Alloy composition was assessed with EDS at various beam energies (Quanta 650 FEG Zeiss Auriga).

### 2.4 Transmission Electron Microscopy

Transmission electron microscopy (TEM JEOL JEM210 200 kV) operated in high-resolution (HRTEM) mode equipped with an electron dispersion spectrometer was used to characterize the morphology of the anodic oxide and Ag NPs. A single nanotube of the anodic layer was analyzed by bending an anodized sample and allowing fractured tubes of the oxide layer to fall on top of a carbon-coated copper grid.

### 2.5 Antibacterial Properties

*E. coli* K-12 was maintained in glycerol stock at −80 °C. For the experimental procedures, bacteria were seeded on lysogeny broth (LB) agar plates overnight at 37 °C; one colony was grown in 4 mL of LB until the stationary phase (overnight at 37 °C). Next, 200 μL of this inoculum was diluted in 20 mL of LB, and bacteria were grown at 37 °C under agitation (150–180 rev/min) until the culture reached an optical density at 600 nm wavelength (OD_600_ = 0.6) corresponding to approximately 2 × 10^8^ CFU (colony forming units)/ml. Metallic samples with an area of 1 cm^2^ were covered with 10 μL of the inoculum (10^8^ CFU/mL) and serial dilutions of 10^7^, 10^6^, and 10^5^ CFU/mL. After inoculation, the samples were incubated for 1 or 3 h at room temperature in Petri dishes within a humid chamber to avoid evaporation of the inoculum. After incubation, the metallic samples were added to assay tubes containing 1 mL of buffered peptone water and submitted to an ultrasound bath for 5 min to detach the bacteria from the sample’s surface. Next, 100 μL of the resultant bacterial suspension was plated onto LB agar and grown overnight under 37 °C for CFU counting. Experiments were performed in duplicate and repeated three times. Statistical analysis was performed using one-way ANOVA (analysis of variance) to compare the means of controls and test groups using the p-value as evidence against the null hypothesis (*p* ≤ 0.05).

## 3 Results and Discussion

### 3.1 Anodic Layer Morphology and Solid-State Precipitation of Ag NPs

**Figure 1:**
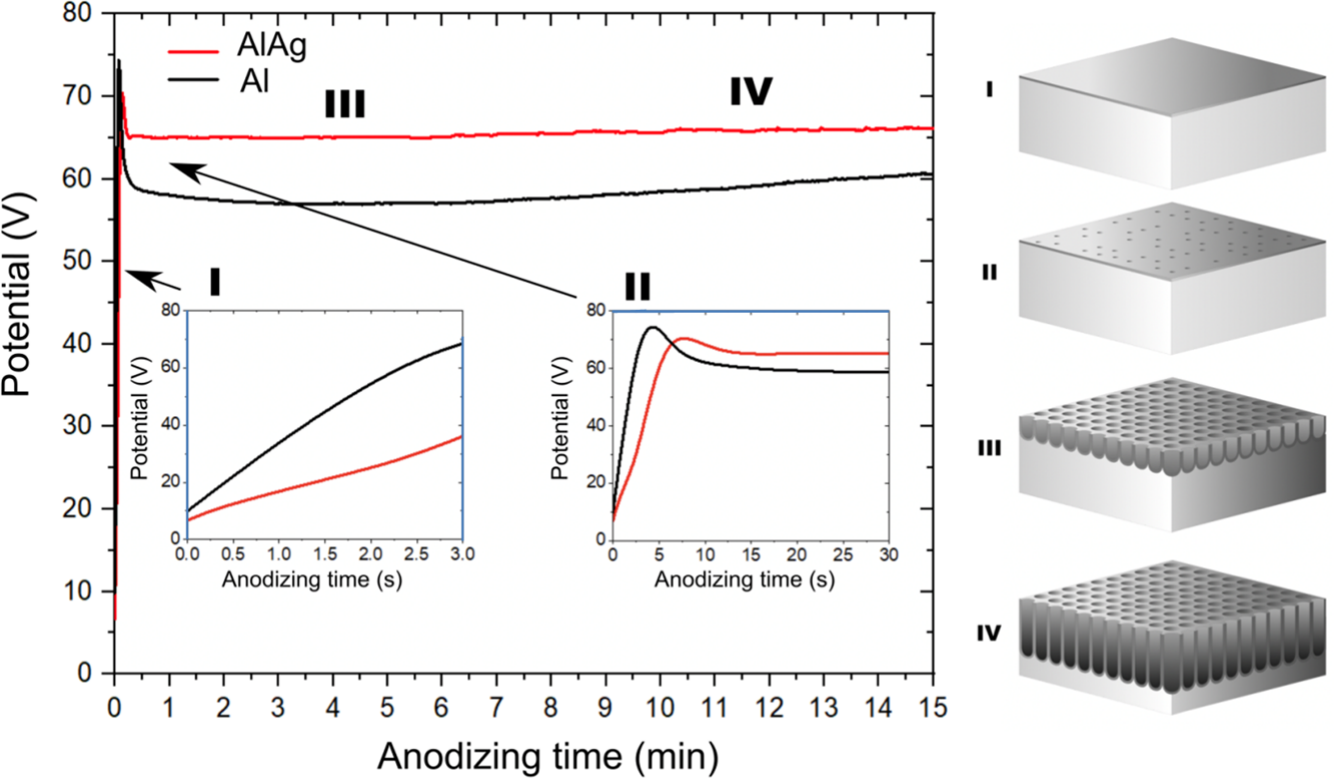
Cell voltage transients (potential vs time) for Al–Ag and pure Al samples during galvanostatic anodizing. The different stages of the anodizing process are illustrated on the right: (I) the barrier oxide layer grows, (II) pitting potential is reached, leading to the initiation of pores, and (III,IV) the pores continue to grow as anodizing progresses.

Figure 1 presents cell voltage transients recorded during the anodization of Al–Ag and pure Al. The usual sequence of events during anodizing is not significantly influenced by alloying Al with Ag, leading to the formation of a porous anodic layer.^19^ During anodizing at a constant current, a compact dielectric Al_2_O_3_ layer (barrier oxide) grew continuously, causing the potential to increase almost linearly (stage I in Figure 1). During our previous work on sulfuric acid anodizing of Al with organic acid additions,^20^ we found that the cell voltage slope (**∂*E***/**∂*t***) in stage I of the anodizing transient (Figure 1) exhibits a linear relationship with the current efficiency of barrier oxide formation, which can be expressed as
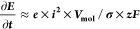
1where ***e*** is the current efficiency of oxide formation, ***i*** is the applied current density, ***V***_**mol**_ is the oxide molar volume, **σ** the ionic conductivity of the oxide, ***z*** is the number of electrons exchanged during the oxidation of Al, and ***F*** is the Faraday constant. Equation 1 is the low-field approximation derived from Ohm’s and Faraday’s laws, as detailed elsewhere.^20^ It is similar to the equation described by Santamaria et al.^21^ The initial magnitude of the electric field across the oxide **∂*E***/**∂*x*** can be estimated considering the thickness of air-formed Al oxide as 3 nm^22^ and an initially applied cell potential of 3 V. Subtracting the potential drop at the inner and outer oxide interfaces, a magnitude close to around 10^–6^ V/cm is obtained, which is usually taken as the limiting **∂*E***/**∂*x*** value of the validity of Ohm’s law.^23^

The initial **∂*E***/**∂*t*** is approximately 23.5 ± 0.5 V/s for pure Al, whereas for the Al–Ag alloy, it was significantly lower at 8.5 ± 0.5 V/s. The nearly 3-fold reduction in the efficiency of oxide formation through alloying with Ag could be attributed to the presence of metallic Ag particles within the oxide, which were electrically connected to the metallic substrate. As a result, a significant portion of the applied anodic current flowed as an electronic current through the interconnected Ag particles in the barrier oxide layer, promoting the oxygen evolution reaction at the Ag/electrolyte interface, where the anodizing slope **∂*E***/**∂*t*** increased to 12.0 ± 0.5 V/s. This initial lower **∂*E***/**∂*t*** and the observed potential arrest have been previously noted by Thompson in Al–Cu alloys^24^ and were attributed to O_2_ evolution on Cu-rich particles embedded within the oxide layer. Similarly, we attributed the increase in anodizing rate and current efficiency after 3 s to the partial loss of electrical interconnectivity between the Ag NPs embedded in the barrier layer and the alloy matrix. This disconnection increased the ionic current fraction of the total applied current, thereby enhancing the current efficiency of the oxide growth.

As anodizing continued, the Al_2_O_3_ layer continued to grow deeper into the bulk of the alloy until the potential at the oxide/electrolyte interface reached the threshold for field-assisted dissolution of the oxide.^25^ Al^3+^ cations were injected into the electrolyte, while Ag remained unoxidized during this process. At this stage (stage II), nanopores nucleated on the oxide surface, leading to a rapid decrease in potential. Initially, these pores grew laterally and then extended toward the metal/oxide interface. Pore formation was accompanied by the partial dissolution of Al_2_O_3_ via field-assisted dissolution, with pores continuing to grow inward. In stage III, the porous oxide layer grew under steady-state conditions: the thickness of the barrier oxide layer remained constant, determining the ohmic drop during pore growth and maintaining consistent pore distribution and electrical resistance (stage III). This steady-state growth continued throughout the subsequent anodizing period (stage IV). Similar to Al–Cu alloys,^24^ alloying Al with the more noble metal Ag increased the potential during stationary porous layer growth, rising from 58 to 60 V for pure Al to 65 V for Al–Ag. This increase was likely due to a lower current efficiency of oxide formation. Ionic current of the oxide formation and electronic current resulting in oxygen evolution flowed parallelly across the oxide and Ag particles of the oxide formation and an electronic current that resulted in the oxygen evolution reaction. Thus, a higher applied potential was necessary to reach the potential of field-assisted dissolution across the oxide at the onset of stage II.

**Figure 2:**
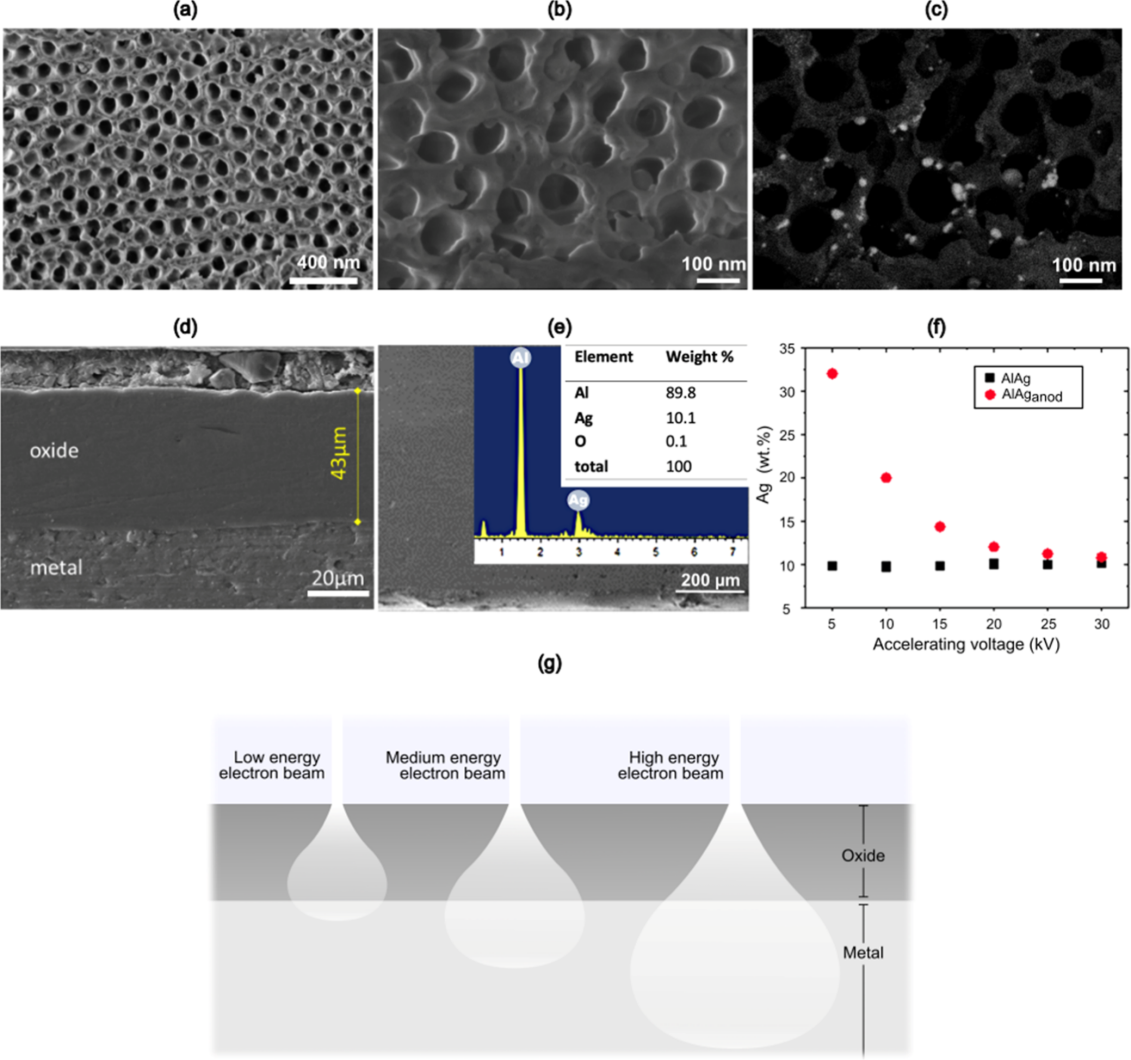
SEM study of anodized Al–Ag: (a) low magnification secondary electron image of anodic layer; (b) high magnification secondary electrons image of anodic layer. (c) High magnification backscattered electron image of anodic layer; (d) traversal section of Al–Ag alloy and anodic layer. (e) EDS for Al–Ag alloy before anodizing. (f) Alloy composition (Ag wt %) as a function of accelerating voltage used in EDS. (g) Illustration of the phenomenon leading to variation in composition as the energy of the electron beam increases.

Scanning electron microscopy (SEM) of anodized Al–Ag samples using secondary electron imaging (SEI) revealed a highly porous surface (Figure 2a,b). The nanopores had a diameter of approximately 100 nm and were considerably uniform in size and pore spacing. Backscattered electron imaging (BSE, Figure 2c) provided atomic density contrast, revealing a dark matrix contrasting with a nanoprecipitate of lighter color due to the larger atomic mass Ag and, consequently, increased elastic scattering. The nanoprecipitate was finely dispersed throughout the matrix and appeared aggregated around the pore edges. A cross-sectional view of the anodic layer shows a homogeneous 43 μm thick oxide layer (Figure 2d). Energy-dispersive X-ray spectroscopy (EDS) analysis of the Al–Ag alloy before anodizing shows a composition of 10 wt % Ag, consistent with the calculated mass ratios for sample preparation (Figure 2e). Interestingly, in the anodized alloy, varying the electron beam accelerating voltage of the EDS analysis (Figure 2f) revealed an increased Ag concentration at lower beam energies, for example, 34 wt % Ag at 5 kV, which decreases as the beam energy increases, eventually matching the composition of the nonanodized sample (10 wt % Ag). For adequate EDS analysis, the electron beam energy should be at least twice the X-ray line excitation energy. For example, for Al Kα (1.48 keV) and Ag Lα (2.98 keV), beam energies of 2.96 keV and 5.96 keV, respectively, would be necessary. Therefore, at a beam energy of 5 keV, some underestimation of the Ag concentration would be expected. Despite this effect, the Ag concentration increased from 10 wt % to approximately 34 wt % as the beam energy decreased from 30 keV to 5 keV. This discrepancy highlights the importance of the EDS sampling depth. According to the Castaing approximation,^26^ the sampling depth ranges from ∼0.3 μm to ∼7 μm as the electron beam energy increases from 5 keV to 30 keV.

Since low-energy electron beams penetrated less deeply into the sample than high-energy beams, the sampling depth for EDS analysis increased with higher beam voltages, thereby increasing the contribution of the substrate to the overall elemental composition (Figure 2g). This suggests that during anodizing, there was a mechanism leading to the concentration of Ag within the nanoporous Al_2_O_3_ layer, which, according to what was found with BSE images and EDS, happened as a Ag-rich phase in the form of NPs.

**Figure 3:**
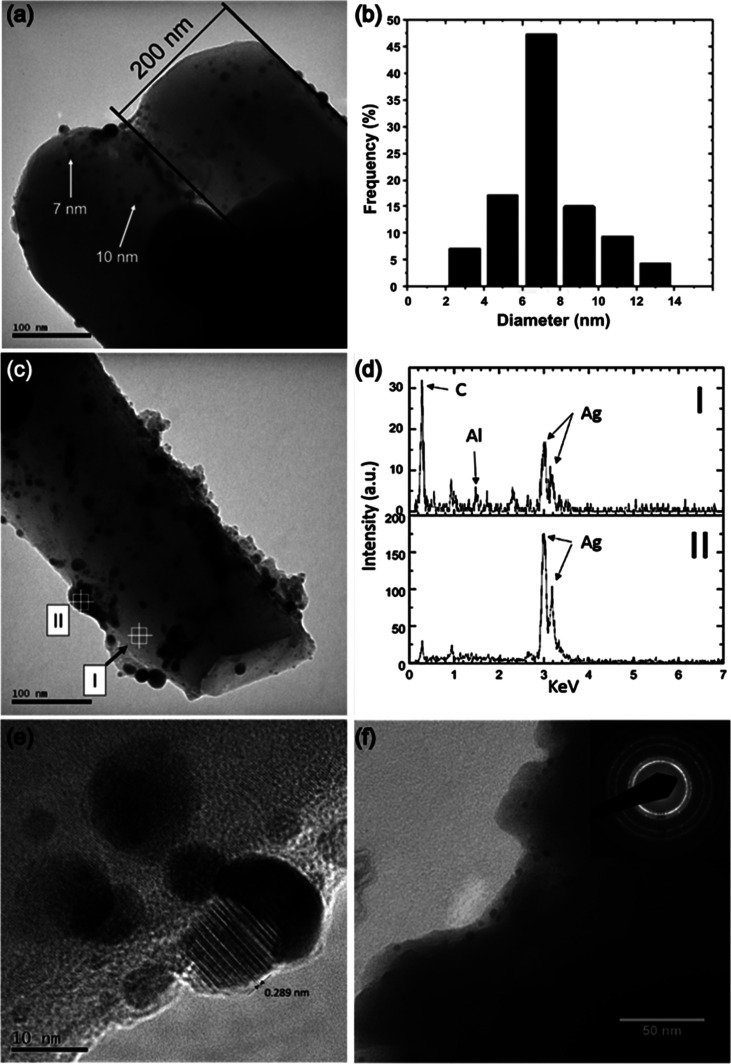
TEM studies: (a) bottom of a unit cell, showing the presence of Ag NPs. Ag NP size distribution from Figure 2a; (c) view of the top portion of a unit cell, showing Ag NPs on its interior (inside the pore) and exterior; (d) EDS from spots I and II in Figure 2c; (e) high-resolution lattice imaging of Ag NPs; and (f) selected area electron diffraction of anodic oxide.

The TEM analysis (Figure 3) of the top and bottom segments of pores detached from the oxide layer gives further information on the anodic layer morphology and composition. The unit cells were nanotube-shaped (Figure 3a,c), and the assembly of these nanotubes created the porous morphology previously discussed (Figure 2). At the base, the unit cell is 200 nm in diameter. A nanocomposite structure was formed by finely dispersed Ag NPs embedded within an Al_2_O_3_ matrix (Figure 3a). The nanoparticles ranged from 2 to 14 nm in diameter, with the majority (70%) measuring up to 8 nm (Figure 3b). The broken portion of a nanotube revealed its interior (Figure 3c). The same composite morphology was observed with nanoparticles homogeneously dispersed inside, outside, and within Al_2_O_3_ (Figure 3c). The EDS patterns shown in Figure 3d from spots I and II are indicated in Figure 3c. The absence of the Al peak in addition to the increase in the Ag peak intensity, when moving electron beam from the matrix region to a precipitate, suggested that the NPs are constituted of pure Ag. The EDS of the matrix (spot I in Figure 3c) also contained Ag, but this could be because Ag NPs were too finely dispersed and abundant not to get caught by the electron beam used for EDS. Further examination at higher magnification and adjustment of focus revealed that the Ag atoms in the NPs were organized in planes, thus being crystalline (Figure 3e). The interplanar spacing of 2.89 Å was found, which is characteristic of (110) planes in face-centered cubic Ag.^27^ Given that anodic Al_2_O_3_ is typically amorphous, the electron diffraction pattern from a larger area of the oxide layer in Figure 3f indicates the presence of randomly oriented crystallites. These findings show that the anodic layer consisted of crystalline Ag NPs embedded within an amorphous Al_2_O_3_ matrix.

A mechanism for forming the morphology observed can be proposed based on the results from Figures 1, 2, and 3. Before anodization, the alloy was heated to dissolve any Ag-rich phases (δ-Ag_2_Al intermetallic) and rapidly cooled to prevent the nucleation of new precipitates. At room temperature, the solubility of Ag in Al is very low,^28^ and the low diffusion rates characteristic of the solid-state hinder the precipitation of new thermodynamically stable phases. However, during the formation of the barrier oxide, vacancy accumulation occurred at the alloy/oxide interface, which diffused into the metal substrate, locally increasing substitutional diffusion and enhancing the mobility of Ag beneath the barrier oxide.^29,30^ With the advancing alloy/oxide interface during anodizing and lack of solubility of Ag in Al_2_O_3_, there was a continuous process of increase in Ag concentration, nucleation into NPs, and incorporation (in the form of NPs) in the barrier oxide, as the metal-oxide interface moved inward. Again, very low diffusion coefficients are involved, and the chemical potential, which is the acting driving force, is lowered by high nucleation rates at the expense of growth.^31^ With the increase in the barrier oxide thickness, the potential of field-assisted dissolution was reached, resulting in pore growth and the porous nanocomposite observed. The reason for the increased particle size near the pore edges at the surface of the anodic layer (Figure 2c) remains unclear. This phenomenon is likely not due to particle growth within the oxide given the low Ag solubility and diffusion rates in Al_2_O_3_. It is more plausible that agglomeration or aggregation occurs during pore formation. However, further studies are needed to describe this phenomenon accurately.

### 3.2 Antibacterial Properties

**Figure 4:**
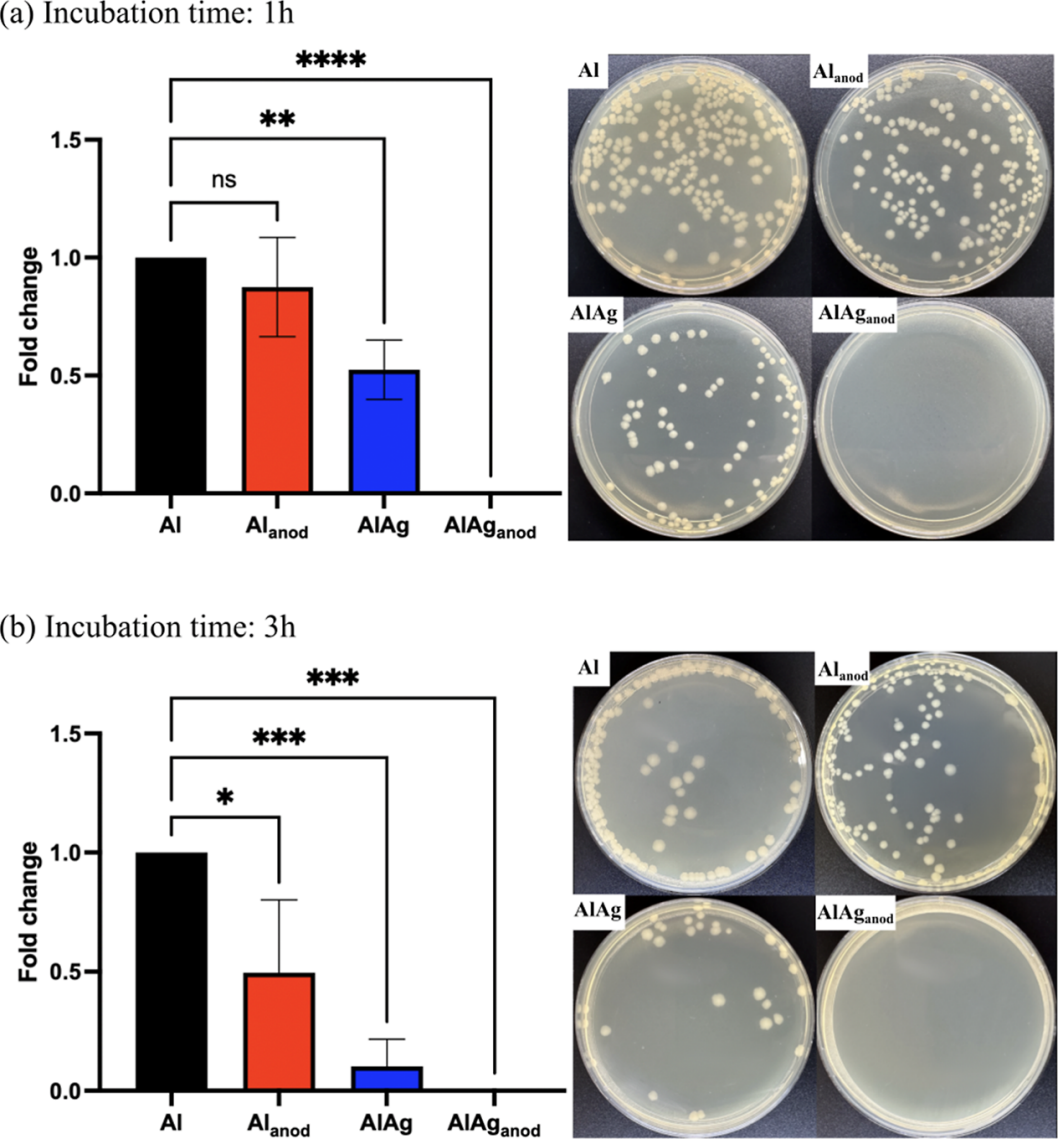
Quantitative analysis of groups submitted to incubation with *E. coli* (K-12) at 10^5^ CFU/mL for (a) 1 h and (b) 3 h. Bar graphs from CFU counting are shown on the top half of the figure as fold change concerning Al. Representatives of LB agar plates used for CFU counting are given in the bottom half of the figure. * (*p* < 0.05), ** (*p* < 0.01), *** (*p* < 0.001), and **** (*p* < 0.0001).

Figure 4 presents CFU counting after 1 and 3 h of incubation for a starting bacterial load of 105 CFU/ml (or 10^3^ CFU/cm^2^). The nonanodized Al–Ag alloy as well as the pure anodized and nonanodized Al were used as controls to assess the effects of anodization and the presence of Ag. The anodized Al–Ag showed no survival of *E. coli* for either incubation time tested, indicating a robust, fast-acting bactericidal effect. Pure nonanodized Al showed no antibacterial properties and was used to normalize other groups. Anodization by itself showed a tendency to increase the antibacterial effect of aluminum, but this effect was only significant after 3 h of incubation, with a 50% reduction in CFU. The incorporation of Ag into nonanodized Al demonstrated a moderate enhancement of antibacterial properties after 1 h, with increased efficacy observed at 3 h. However, all samples in this group showed considerable corrosion by pitting across the entire tested surface due to the presence of Cl^–^ in PBS used to prepare *E. coli* dilutions. The observed bactericidal effect in nonanodized Al–Ag samples was likely due to this destructive corrosion process, potentially augmented by the precipitation of Ag-rich phases.

In contrast, all other experimental groups remained structurally unaffected by incubation with *E. coli* in PBS. Considering the destructive effects of the bacterial testing on nonanodized Al–Ag, this group was not used in further experiments. The bottom half portion of Figure 4 is used for a representative CFU counting on LB agar plates.

**Figure 5:**
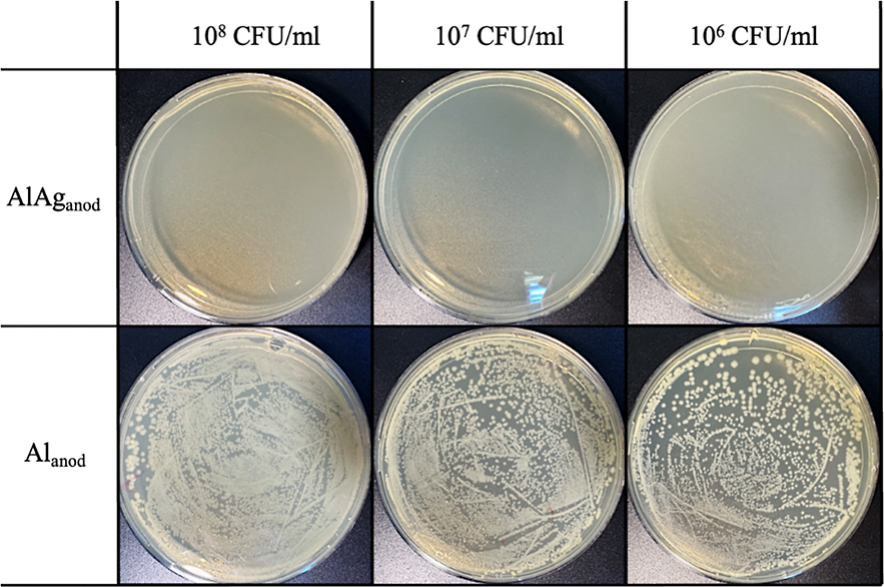
Qualitative assays showing the effect of the presence of Ag at 10 wt % in anodized samples after incubation with 10^8^, 10^7^, and 10^6^ CFU/mL of *E. coli* for 3 h.

The samples were then submitted to high bacterial loads to test the limits of the anodized Al–Ag bactericide effect (Figure 5). *E. coli* dilutions with 10^8^ CFU/mL (10^6^ CFU/cm^2^), 10^7^ CFU/mL (10^5^ CFU/cm^2^), and 10^6^ CFU/mL (10^4^ CFU/cm^2^) were incubated for 3 h. Since the objective was to analyze the effect of Ag NPs, only the anodized groups were used. Again, anodization under the parameters employed resulted in a robust bactericide effect when silver was present. While CFU was too numerous for counting on anodized Al, no bacteria survived the incubation on anodized Al–Ag, even at 10^8^ CFU/mL.

The anodic layer developed benefits from a few characteristics of high interest in antibacterial applications. First, the anodic layer is high in Ag NP content. Ag NPs are excellent antibacterial materials, with their bactericide effect being a consequence of Ag^+^ interaction with bacterial DNA, membranes, and proteins.^5^ The Ag NPs synthesized are in the range of 2–14 nm, which is optimal to inhibit bacterial activity.^32−34^ Also, the Ag NPs are well dispersed within the Al_2_O_3_ matrix and cannot be detached from the matrix. These characteristics are all a consequence of the solid-state nucleation during the synthesis process developed, which occurs under low diffusion rates with vacancies created in close proximity to the moving oxide/metal interface, working as nucleation sites for Ag NPs (see discussion of Figures 1–3).

**Figure 6:**
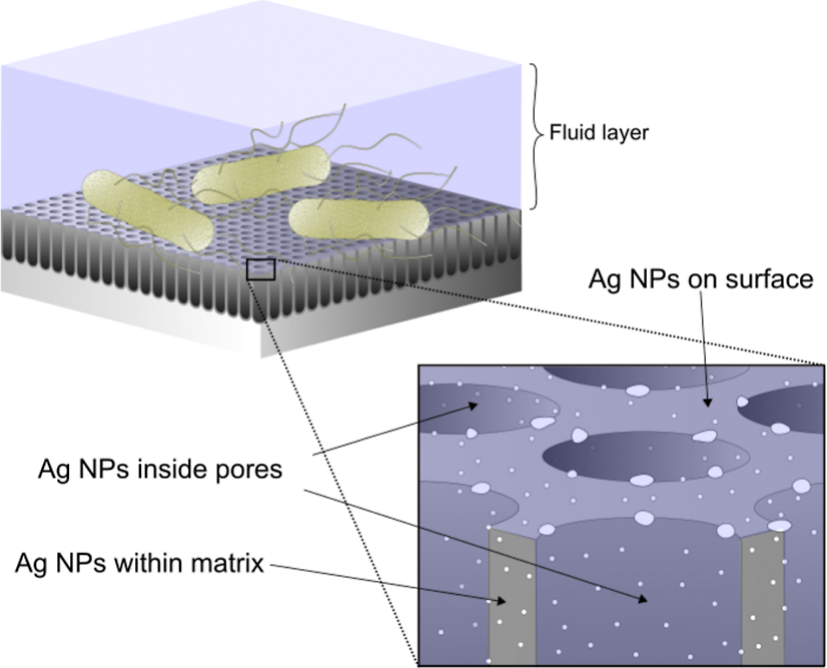
Illustration of bacterial interaction with the anodic layer containing AgNPs synthesized in the solid state during anodizing. Zoomed region highlights the presence of Ag NPs as part of a porous composite layer.

A distinctive feature of this anodic layer is the multilevel exposure of Ag NPs to the environment and, consequently, bacteria (zoomed region in Figure 6). There are Ag NPs exposed on top of the anodic layer, inside pores, and within the Al_2_O_3_ layer. Ag NPs exposed on top of the anodic layer are readily accessible to bacteria and could exhibit a stronger bactericidal effect. The bactericidal effect of Ag NPs is believed to result from the generation of reactive oxygen species (e.g., H_2_O_2_) during Ag^+^ release.^35^ Ag^+^ ions, which have a high affinity for thiols in biomolecules, such as proteins and enzymes, form stable complexes that deactivate critical metabolic and respiratory pathways, ultimately leading to cell death. ROS induce oxidative stress, which damages cellular components, including lipids, proteins, and nucleic acids.^35^

The mechanisms governing the release of Ag^+^ from Ag NPs within pores remain uncertain. However, this effect could be beneficial in scenarios where biological fluids carrying bacteria penetrate the pores, triggering Ag^+^ release, or during cleaning with aqueous solutions. In such cases, the presence of Ag NPs within nanopores may play a critical role in maintaining antibacterial properties through sustained Ag^+^ release.^9^ Furthermore, the Ag NPs embedded within the oxide matrix offer the potential for long-lasting antibacterial activity, particularly in the event of surface damage. Damage to the anodic layer could expose additional Ag NPs, enhancing their antibacterial effect. While this material is not intended for orthopedic implants, as aluminum is rarely used in such applications, embedding Ag NPs within the matrix improves its safety profile by preventing the release of nanoparticles into the environment.^33^

Nanotopographies, as observed in this study, have been reported to have antibacterial properties due to physical disruption of the bacterial cell wall independent of biochemical interactions with functionalities on the material surface.^15,16,36^ Several mechanisms are proposed as responsible for bacterial cell death, and they are probably influenced by multiple factors, such as cell wall structure, bacteria mobility, and specific features of the nanostructure.^15,37^ Most authors agree that a mechanism dependent on cell wall deformation due to adhesive forces leads to the stretching and rupturing of the cell wall in the valleys between surface features.^11,13,38,39^ In addition to these effects, a reduced capacity of bacteria to replicate on nanostructured surfaces, punctures of the cell wall, and induction of oxidative stress are proposed as mediators of antibacterial effects.^14^ While previous studies focused on physical mechanisms due to surfaces with nanopillars, nanopores probably rely on similar mechanisms.^15,17^ For the specific case of nanopores on metallic surfaces, an increase in hydrophilicity caused by the nanotopography was observed to be a major factor in decreasing adhesion and the ability to proliferate of *E. coli*, *Pseudomonas aeruginosa*, and *Staphylococcus aureus*, as the bacterial cell wall is somewhat hydrophobic.^16,36^ It is important to note that the antibacterial properties in this study were confirmed only for Gram-negative bacteria. Antibacterial properties for other bacterium types such as Gram-positive or biofilm-forming would demand a reassessment of antibacterial properties. In the case of incomplete elimination of bacteria, increased concentrations of Ag in the alloy or longer incubation times could be tested.

Given that an increase in the antibacterial effect for the controls was observed from 1 to 3 h of incubation (Figure 4), a further increase in incubation time would likely generate additional antibacterial action. However, incubation times tested were not increased due to complete elimination of *E. coli* after 1 h on the anodized AlAg. Studies that found more significant CFU reductions on pure Al utilized extended incubation periods (24 h)^16,36^ or incubation under dry conditions.^17^ While dry conditions more closely mimic real-world applications, they are very challenging for bacterial survival.^40^ From the results presented here (Figures 4 and 5), the nanotopography is likely acting as an adjuvant, while Ag NPs are the main factor responsible for the antibacterial properties of the anodic layer.

## 4 Conclusions

In this study, we have successfully developed a method to synthesize Ag NPs from Al–Ag alloys by anodizing, creating a nanoporous structure that combines physical and chemical antibacterial mechanisms. Compared with current methods that incorporate Ag NPs on top of nanopores as a second deposition process, this method is unique because it is capable of synthesizing Ag NPs not only on the surface of the material but also inside pores and within the Al_2_O_3_ matrix in only one step. We demonstrated that this method could produce small, well-dispersed Ag NPs (2–14 nm) in the nanoporous matrix due to precipitation in the solid state. Furthermore, the porous oxide layer eliminated *E. coli* with up to 10^8^ CFU/mL under 3 h, possibly by combining the physical antibacterial mechanism of the nanotopography with the chemical bactericide effect of Ag NPs. The nanotopography fabricated with a nanocomposite structure creates the potential for long-lasting antibacterial properties, as damage to the surface would expose additional Ag NPs. This innovative strategy is promising for any application that would benefit from surfaces with durable and robust antibacterial effects such as high-touch surfaces in the hospital setting.
